# Early follow-up blood cultures—frequency and outcomes of repeat blood culture collection within 48 hours of emergency department workup: an observational study

**DOI:** 10.1017/ash.2025.10085

**Published:** 2025-09-05

**Authors:** Angela Zara Hills, Jaimi Greenslade, Mercedes Ray, Julian Williams

**Affiliations:** 1 Emergency and Trauma Centre, Royal Brisbane and Women’s Hospital, Brisbane, Australia; 2 Faculty of Health, Queensland University of Technology, Brisbane, Australia; 3 Faculty of Medicine, University of Queensland, Brisbane, Australia

## Abstract

**Objective:**

To determine the frequency and outcomes of early follow-up blood cultures (BCs) collected within 48 hours of patients being investigated for bacteremia in the emergency department (ED), as well as the number of new pathogens isolated.

**Design:**

Retrospective observational study of patients who had BCs collected in the ED between October 2019 and July 2020.

**Methods:**

This study was conducted in a large, metropolitan ED with annual census of over 82,000 adult presentations. ED patients who had BCs collected during the study period were identified, and those who had BCs recollected within 48 hours were identified as having early follow-up BCs. The characteristics of these patients were compared to those without early follow-up BC collection. Logistic regression analyses were conducted to determine relationships between specific pathogens in EDBCs and early follow-up BC collection.

**Results:**

During the study period, 68,330 patients were treated in the ED, and BCs were collected from 1821 (2.7%). Of these, 449 (24.7%) had BCs recollected within 48 hours of their initial ED workup (early follow-up BCs) and were re-cultured 789 times across their collective stays. Five patients (1.1%) grew pathogens not isolated in EDBCs, all of which were susceptible to concurrent antimicrobials. No new pathogens were isolated in BCs taken >48 hours post ED workup.

**Conclusions:**

Collection of early follow-up BCs was common. However, the rate of new pathogen growth was low and contributed minimally to patient management. Given associated costs and patient discomfort, the practice should be discouraged unless to clarify potential false positive results in ED BC.

## Introduction

Blood cultures (BCs) remain the primary method for diagnosing bacteremia and can assist when targeting antibiotic therapy. Collection of quality BCs in the emergency department (ED) prior to commencement of antimicrobial therapy optimizes likelihood of isolating a true pathogen.^1–3^ BC results can take up to five days, and frequently there is no pathogen growth. The value of subsequent BCs collected within 48 hours of initial workup and commencement of antimicrobial therapy remains unclear, however this practice often occurs in response to fevers or other markers of unwellness.^4^ BCs are routinely collected from two separate collection sites, thus each subsequent BC could entail two or more instances of venepuncture for patients who may have already been subjected to numerous invasive procedures.^5^ Moreover, although exact costs are beyond the scope of this inquiry, repeated collection and processing of BCs is a needless economic burden on the healthcare system if these tests add little to the clinical picture.

Numerous studies have examined the frequency and impact of follow-up BCs. Some focus on individual organisms such as Staphylococcus aureus,^6–8^ Streptococcus^9^ and Pseudomonas aeruginosa,^10^ or more broadly on Gram negative bacteremia.^11–16^ Others have reviewed the incidence of follow-up BCs across the spectrum of pathogens causing bacteremia.^17,18^ Some authors opted to investigate BCs and bacteremia in discrete illnesses such as febrile neutropenia.^19,20^ To our knowledge, only one inquiry has included non-bacteremic patients without limiting enrollment based on comorbidities,^4^ and no previous authors have focussed on patients who have initial BCs collected in ED. Rather, previously studied cohorts had index BCs collected at random points in their hospital stay, and repeated collection 24 hours to 7 days subsequently.^15,16,19,21^

The utility of BCs collected within 48 hours of ED BC collection remains unexamined, and it was patients who have BC collection repeated in this time frame (early follow-up BCs) that were the subject of our inquiry. Investigating the incidence and outcomes of early follow-up BCs in all patients regardless of bacteremic status is vital.

Our inquiry aimed to determine (1) the incidence of early follow-up BCs and the number of BCs collected from these patients in total during their hospital stays, (2) the proportion of early follow-up BCs that grew a new pathogen and whether this necessitated a change in treatment, and (3) the clinical, physiological and microbiological factors associated with early follow-up BC collection, persistent bacteremia, and new pathogen growth. We hypothesized that BCs added little value to patient care when repeated within 48 hours of ED BC collection.

## Methods

### Study design and setting

This retrospective observational study was conducted in the ED of a large tertiary adult hospital in Brisbane, Australia, with an annual census over 82,000 presentations. This study was approved by the local research ethics committee, which deemed patient consent was not required (HREC/2021/QRBW/75053).

### Participants and data sources

All patients who had BCs collected in the ED from October 2019 to July 2020 were identified using the hospital’s pathology information system. Demographic, physiological, and microbiological data were entered into a study database (Access, Microsoft, WA). Early follow-up BCs were defined as BCs collected within 48 hours of index BC collection in the ED.

For patients with early follow-up BCs, additional data were abstracted and entered into a password-protected database.^22^ All instances of BC collection within 48 hours of a previous set were recorded, up to a maximum of eight repeat collections. Collection time, triggers, and whether the BC was a single or paired set was recorded, as accepted procedure dictates that BCs are best taken in pairs from two separate puncture sites.^5^ We considered sets to be “paired” if they were collected within two hours of each other.

All BC results were determined to be true positive (bacteremia), false positive (contaminated), or negative. A true positive BC was defined as growth of a pathogen in one or more BC bottles (S. aureus, S. lugdunensis, S. pneumoniae, beta-hemolytic Streptococci, Enterococci, Gram-negative rods and fungi were considered pathogens). Cultures that grew generally accepted contaminants (coagulase negative Staphylococci, Bacillus sp. [except anthrax], Corynebacterium sp., S. viridians, Micrococcus sp., Cutibacterium acnes and Diphtheroids sp.) were considered false positive unless organisms were isolated from multiple or repeated BCs.^23,24^ The results reported by the microbiology team were verified through clinical correlation, documentation by the treating team and/or the infectious disease team, and discussion with a clinical expert where there was equivocation about the significance of the result.

Early follow-up BC collection initiated chart reviews by an experienced clinician to determine the trigger/rationale for re-investigation, including fever, confirmation of a previous true or false positive BC result, clinical deterioration, and “routine” (“daily” or “surveillance” BCs documented as part of the treating team’s management plan). Where the trigger was not obvious from clinical notes or vital observations an “unknown trigger” was allocated.

### Microbiological methods

All BCs were collected in standard BacT/ALERT® FA PLUS and FN PLUS bottles, and incubated with the BacT/Alert® Virtuo (bioMerieux, France) system for five days. Samples from positive culture bottles were Gram stained, plated and further incubated for 48 hours. Pathogens growing on plates underwent species identification with MALDI-TOF MS (VITEK MS, bioMerieux, France) and susceptibility testing with VITEK 2 (bioMerieux, France).

### Statistical methods

Baseline characteristics of the study participants were presented for the overall cohort, and for those with and without early follow-up BCs. The absolute difference between study groups and 95% confidence intervals of the difference were calculated. The results of BC testing were determined, including the number of negative, true positive and false positive BCs. Two sets of logistic regression analyses were conducted to identify if there was a relationship between the causative pathogen identified in ED BCs and early follow-up BCs. The first considered all patients, including those with no pathogen grown, while the second included only those patients with a positive ED BC. The recorded rationale for collecting early follow-up BCs were reported for various groups, including all patients, those who only had one set of ED BCs, patients with a new pathogen identified in early follow-up BCs, and categorized by the outcomes of early follow-up BC collection (positive, negative, or contaminated).

## Results

**Figure 1.**
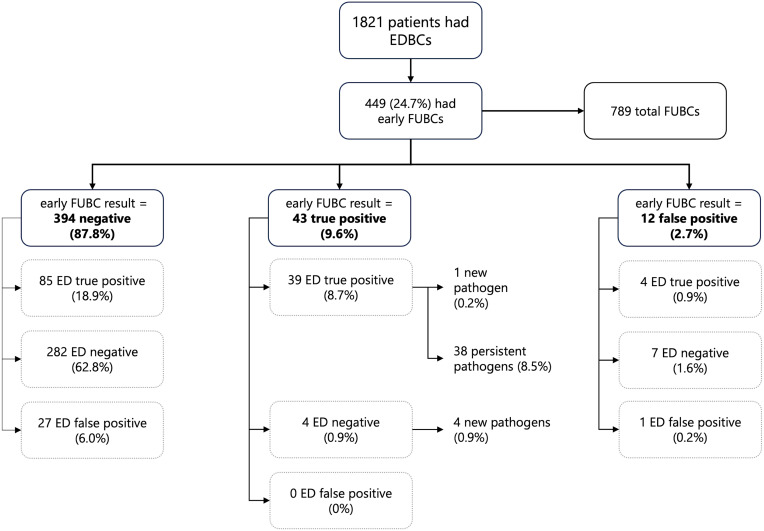
Study cohort and blood culture results.

Over the study period (October 2019–July 2020), 68,330 patients were treated in the ED and 1821 (2.7%) underwent BCs (Figure 1). Of these, 449 (24.7%) had BCs recollected within 48 hours of their initial ED workup (early follow-up BCs). These patients went on to be re-cultured 789 times across their collective stays.

### Cohort characteristics

Patients who had early follow-up BC collection comprised an older and sicker cohort than those who did not (Table 1). The median age for patients with and without early follow-up BCs collected was 55 (interquartile range [IQR] 37–72) and 60 (IQR 45–72). A greater proportion of those with early follow-up BCs received care in the intensive care unit (9.2%), compared to 4% of patients with no early follow-up BC collection. A higher proportion of the early follow-up BC group were immunosuppressed (36.3% vs 25.2%) and/or were neutropenic (8.9% vs 3.5%). The early follow-up BC group had a higher Mortality in Emergency Department Sepsis score (8 [IQR 3–12] vs 6 [IQR 3–9]), Charlson Comorbidity Index (4 [IQR 2–6] vs 3 [IQR 0–6]) and total Sequential Organ Failure Assessment score (2 [IQR 1–4 vs 1 [IQR 0–3]). Despite this, there was no statistical difference in mortality at either 48 hours or 30 days between the groups.

**Table 1.** Characteristics of study cohort

Characteristics	Entire cohort (*n* = 1821)	Patients without repeat BCs (*n* = 1 372)	Patients with repeat BCs (*n* = 449)	Difference (95% CI)
Age	57 (38.5–72)	55 (37–72)	60 (45–72)	**5 (1.5 to 8.5)**
Female Sex	740 (40.7%)	565 (41.2%)	175 (39.0%)	−2.2% (−7.4 to 3.0%)
RACF	98 (5.4%)	73 (5.3%)	25 (5.6%)	0.2% (−2.2 to 2.7%)
Initial SBP	128 (114–145)	129 (114.5–143)	128 (112–145)	−1 (−1.2 to 3.2)
Immunosuppression^i^	509 (28.0%)	346 (25.2%)	163 (36.3%)	**11.1% (6.1 to 16.1%)**
Eligible for ICU	1,495 (82.1%)	1,131 (82.4%)	364 (81.1%)	−1.4% (−5.5 to 2.8%)
Time to effective Abs, h	2.8 (1.7–5.5)	2.7 (1.6–5.2)	2.9 (1.7–6.2)	0.2 (−0.2 to 0.5)
ED Length of Stay, h	6.0 (4.1–9.1)	5.7 (4.0–8.9)	6.5 (4.6–10.0)	**0.8 (0.4 to 1.3)**
Hospital Length of Stay, days	4 (1–7)	3 (1–6)	6 (4–11)	**3 (2.5 to 3.5)**
Death within 48 hours	22 (1.2%)	19 (1.4%)	3 (0.7%)	−0.7% (−1.7 to 0.3)
Death within 30 days	98 (5.4%)	72 (5.2%)	26 (5.8%)	0.5% (−1.9 to 3.0%)
Disposition				
ICU admission	96 (5.3%)	54 (4.0%)	41 (9.2%)	**5.1% (2.3 to 8.0%)**
Ward	1,521 (83.5%)	1,116 (81.3%)	405 (90.2%)	**8.9% (5.4 to 12.3%)**
Died in ED	8 (0.4%)	8 (0.6%)	0 (0.0%)	-0.6% (−1.0 to −0.0)
Home	196 (10.8%)	193 (14.1%)	3 (0.7%)	**−13.4% (−15.4 to −11.4%)**
Source				
Respiratory	377 (20.8%)	296 (21.7%)	81 (18.0%)	−3.6% (−7.8 to 0.5%)
Urinary tract infection	282 (15.5%)	202 (14.8%)	80 (17.8%)	3.0% (−1.0 to 7.0%)
Abdo/Pelvic	222 (12.2%)	161 (11.8%)	61 (13.6%)	1.8% (−1.8 to 5.4%)
Soft tissue	265 (14.6%)	202 (14.8%)	63 (14.0%)	−0.8% (−4.5 to 3.0%)
Other	101 (5.6%)	53 (3.9%)	48 (10.7%)	**6.8% (3.8 to 9.8%)**
Unknown^ii^	568 (31.3%)	452 (33.1%)	116 (25.8%)	**−7.3% (−12.1 to −2.5%)**
ED bloods				
Lactate	1.7 (1.2–2.9)	1.8 (1.2–3)	1.7 (1.2–2.7)	−0.1 (−0.3 to 0.1)
Albumin	36 (32–40)	36 (32–40)	34 (30–38)	**−2 (−3.1 to −0.9)**
White cell count	10 (6.6–14.4)	9.9 (6.7–14.2)	10.6 (5.9–15.4)	0.7 (−0.8 to 1.4)
Neutropenia^iii^	88 (4.8%)	48 (3.5%)	40 (8.9%)	**5.4% (2.6 to 8.2%)**
True positive ED BC	223 (12.2%)	95 (6.9%)	128 (28.5%)	**21.6% (17.2–26.0%)**
False positive ED BC	38 (2.1%)	10 (0.7%)	28 (6.2%)	**5.5% (3.2 to 7.8%)**
Single Set taken in ED	358 (19.7%)	295 (21.5%)	63 (14.0%)	**−7.5% (−11.4 to −3.6%)**
Scores (medians)				
MEDS	6 (3–9)	6 (3–9)	8 (3–12)	**2 (0.9 to 3.1)**
CCI	3 (1–6)	3 (0–6)	4 (2–6)	**1 (0.5 to 1.5)**
SIRS	2 (1–3)	2 (1–3)	2 (2–3)	0 (−0.5 to 0.5)
SOFA	2 (0–3)	1 (0–3)	2 (1–4)	**1 (0.3 to 1.7)**

Data are presented as median (IQR) for continuous measures, and n (%) for categorical measures. RACF, residential aged care facility; SBP, systolic blood pressure; ICU, intensive care unit; Abs, antibiotics; ED, emergency department; BC, blood culture; MEDS, mortality in emergency department sepsis; CCI, Charlson comorbidity index; SIRS, systemic inflammatory response syndrome; SOFA, Sequential organ failure assessment.

i Chemotherapy, radiation therapy, daily steroid (not inhaled), posttransplant, or other immunomodulation.

ii Includes primary bacteremia, fungaemia, and pyrexia of unknown origin.

iii Neutrophil count <.5x10 ^ 9/L.

### New pathogen growth

Five patients who had early follow-up BCs collected (1.1%) grew a pathogen that was not isolated in ED BCs (Figure 1). However, culture specimens (other than blood) collected within 24 hours of presentation identified these causative pathogens in four cases (three ED urine specimens, and one sputum specimen collected in ICU). All new pathogens were Gram negative (three *Klebsiella pneumoniae* and two *E. coli*), and all were sensitive to the antimicrobial therapy being administered when the result was returned.

No additional BCs collected after the first repeat grew new pathogens. The new pathogen rate of all BCs collected for this patient cohort was therefore 0.6% (5/789).

### Pathogens

**Figure 2.**
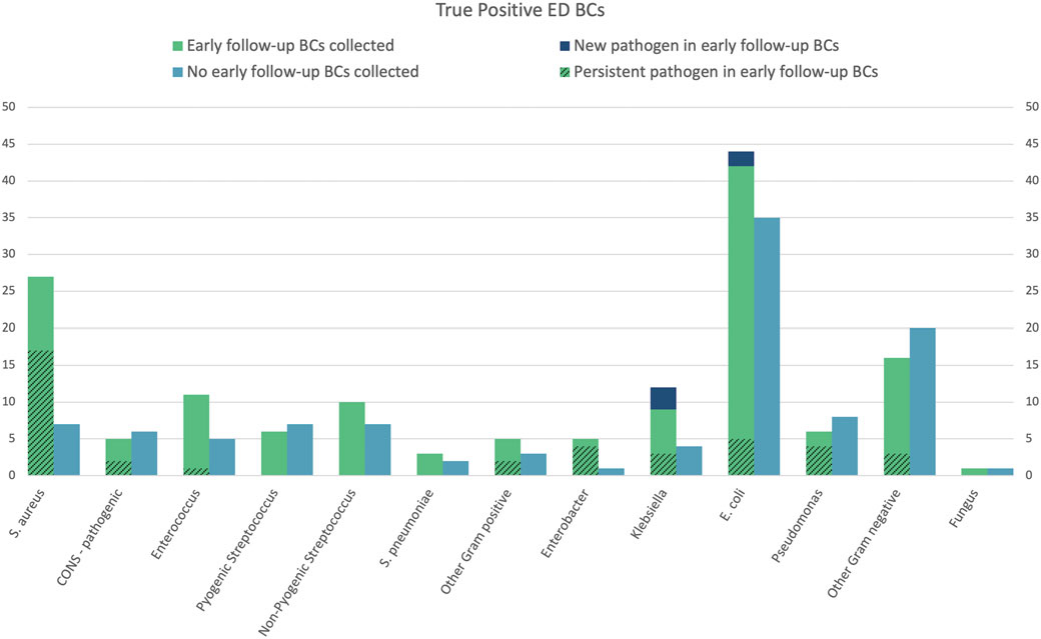
Pathogen growth in EDBCs, persistent and new pathogen growth in early follow-up blood cultures. CONS = coagulase negative staphylococcus.

Pathogens identified in ED BCs and early follow-up BCs are shown in Figure 2. *S. aureus* was the most common cause of persistent bacteremia, although *Enterobacter* and *Pseudomonas* had greater rates of persistence in early follow-up BCs. Despite this high proportion of persistence most patients (57.1%) with *Pseudomonas* growth in their ED BCs were not recultured within 48 hours.

Bacteremic patients who were in hospital for >48 hours and not recultured had either Gram-negative pathogens, or non-pyogenic *Streptococcus* isolated in their ED BCs. Patients who grew *S. aureus* or pathogenic coagulase negative *Staphylococcus* in ED BCs and were not recultured within 48 hours were likely to have been discharged (either facilitated or against medical advice) or died within 48 hours. Instances of recall for treatment have not been investigated here.

Gram-positive pathogens (*n* = 104) were recultured in 64.4% of cases, and Gram-negative pathogens (*n* = 147) in 53.1% of cases. Table 2 details the likelihood of specific pathogens being associated with early follow-up BCs.

**Table 2.** EDBC pathogens associated with early follow-up blood culture collection

Isolated Species	Total (*n* = 1850)	No repeat culture within 48 hours (*n* = 1 384)	Repeat culture within 48 hours (*n* = 466)	OR^*^ for the entire cohort (95% CI)	OR^**^ for BC pos patients only (95% CI)
S. aureus	34 (1.8%)	7 (20.6%)	27 (79.4%)	**12.1 (5.2 to 28.0)**	**3.3 (1.3 to 7.9)**
Coagulase negative staphyloccus—pathogenic	11 (0.6%)	6 (54.6%)	5 (45.5%)	2.5 (0.6 to 11.2)	0.6 (0.1 to 2.8)
Enterococcus	16 (0.9%)	6 (37.5%)	10 (62.5%)	**5.0 (1.8 to 13.8)**	1.2 (0.5 to 3.4)
Pyogenic Strep	13 (0.7%)	7 (53.9%)	6 (46.2%)	2.6 (0.9 to 7.7)	0.6 (0.2 to 1.9)
Non-Pyogenic Strep	17 (0.9%)	7 (41.2%)	10 (58.8%)	**4.3 (1.6 to 11.4)**	1.1 (0.4 to 2.9)
Strep pneumoniae	5 (0.3%)	2 (40%)	3 (60%)	4.5 (0.7 to 26.9)	1.1 (0.2 to 6.8)
Other gram positive	8 (0.4%)	3 (37.5%)	5 (62.5%)	**5.0 (1.2 to 20.8)**	1.2 (0.3 to 5.2)
Enterobacter	6 (0.3%)	1 (16.7%)	5 (83.3%)	**15 (1.8 to 128.6)**	3.7 (0.4 to 32.5)
Klebsiella	13 (0.7%)	4 (30.8%)	9 (69.2%)	**6.8 (2.1 to 22.1)**	1.7 (0.5 to 5.5)
E. coli	77 (4.2%)	35 (45.5%)	42 (54.5%)	**3.8 (2.4 to 6.0)**	0.8 (0.5 to 1.4)
P. aeruginosa	14 (0.8%)	8 (57.1%)	6 (42.9%)	2.2 (0.8 to 6.5)	0.5 (0.2 to 1.6)
Other gram negative	36 (2.0%)	20 (55.6%)	16 (44.4%)	**2.4 (1.2 to 4.8)**	0.5 (0.3 to 1.1)
Fungus	2 (0.1%)	1 (50%)	1 (50%)	3.0 (0.2 to 47.7)	0.7 (0.0 to 12)
No causative pathogen^***^	1597 (86.3%)	1 277 (79.9%)	321 (20.1%)	**0.2 (0.1 to 0.3)**	

N.B. Some BCs grew multiple organisms, therefore the number of pathogens is greater than the number of patients in this cohort.

OR, odds ratio.

* odds of each category triggering a repeat culture compared to any other result.

** odds of each category triggering a repeat culture compared to any other positive result.

*** organisms determined to be contaminants are included in this group.

Gram-negative bacteremia persisted beyond a second repeat BC in two cases of *Escherichia coli*. One of these patients had three instances of BC collection within 24 hours of ED workup (all three were positive for the same pathogen). Further BCs were not collected to show bacteremia clearance. The second patient failed to receive source control in a timely manner due to an unidentified cause of their bacteremia. After bacteremia clearance was shown, the patient went on to have three additional BC collections.

### ED BC results and decision to re-culture

Of the 449 patients who had early follow-up BCs collected, 293 (65.3%) had negative ED BCs. These patients went on to have 315 BC collections in addition to those collected in ED.

Ninety–four patients grew a pathogen in their ED BCs and were not subsequently recultured in the following 48 hours. Twenty (21.3%) of these patients were discharged within this time and a further seven (7.4%) died. Sixty–three (67%) of these patients (including those who died or were discharged) were not recultured at all during their stay, 7 (7.4%) were recultured within 48–72 hours of ED workup, and 24 (25.5%) were recultured sometime after 72 hours.

### Blood culture quality

The rate of single set BC collection within the ED was 19.7%, compared to 73.8% for BCs collected subsequent to this. False positive rates in ED BCs (2.1%) was similar to early follow-up BCs (2.7%). Early follow-up BCs were taken in 28 patients with false positive ED BCs, returning a negative result in 27 (96%).

### Triggers

Triggers for BCs collection after ED workup were classified into five groups (Table 3). Repeat BC collection was most likely to be requested as part of “routine” blood collection (48.3%). Most new pathogens were grown in the context of fever (*n* = 3). Justification for repeating BCs was recorded 788 of the 789 cases (99.9%).

**Table 3.** Triggers for follow-up blood culture collection

Triggers	All repeat cultures (*n* = 789)*	Single Set (*n* = 582)	True Positive (*n* = 68)**	False Positive (*n* = 13) **	Negative (*n* = 708) **	New Pathogen grown**
Routine^***^	381 (48.3%)	303 (79.5%)	29 (7.6%)	8 (2.1%)	344 (90.3%)	1 (0.3%)
MERT/deterioration	60 (7.6%)	45 (75.0%)	3 (5%)	0 (0%)	57 (95%)	1 (1.7%)
Fever	293 (37.1%)	199 (67.9%)	20 (6.8%)	4 (1.4%)	269 (91.8%)	3 (1.0%)
Confirmation of result	80 (10.1%)	51 (63.8%)	21 (26.3%)	1 (1.3%)	58 (72.5%)	0 (0%)
Unknown	1 (0.1%)	0 (0%)	0 (0%)	0 (0%)	1 (100%)	0 (0%)

* Data are n (column percentage). There may be multiple triggers for repeat cultures so column % will not equal 100%.

** Data are n (row percentage)

*** Either “daily” or “surveillance” BCs documented in the patient chart as part of the treating team’s management plan and BC request included on basic blood form alongside other blood tests

MERT, medical emergency response team.

Early follow-up BC collection with a “routine” trigger was most often performed by a phlebotomist, whereas collection due to deterioration/activation of an emergency response (MERT) or fever was most often performed by medical or nursing staff.

## Discussion

Out of 1,821 patients who had ED BCs collected during the study period, 449 (26.7%) had early follow-up BCs collected. Of these, 5 (1.1%) grew a pathogen not isolated in ED BCs. None of these patients required alterations to established care to account for the new pathogen. No new pathogens were isolated from any patient beyond the first set of repeated BCs. 293 patients (65.3%) did not grow any pathogen in ED BCs and these patients were re-cultured a total of 315 times.

A high rate of BC recollection may be associated with significant cost to the healthcare system for little positive yield. Each instance of BC collection represents material consumption, collecting clinician time, processing by pathology staff, occupation of space in an incubator, and eventual consideration of the result by the treating team. Patient discomfort due to repeated invasive procedures is also a concern, with each venepuncture increasing the risk of complication and impacting their experience.

Our data are retrospective and observational, and decisions to collect early follow-up BCs were made by clinicians. The data thus reflect clinician practices in response to patient diagnosis and physiology rather than a comparison of patients randomly assigned to either have early follow-up BCs collected or not. Additionally, data were limited to those available on chart review.

Examination of BCs collected within 48 hours of ED BC collection differs from existing literature, thus limiting comparability of results. While patients who had BCs repeated >48 hours post ED workup were noted, comprehensive demographic or physiological data were not collected.

Although our study is limited to patients who had ED BCs collected, we sought to keep the cohort broad by including patients with and without proven bacteremia. The 2004 inquiry by Tabriz *et al.* examined bacteremic and non-bacteremic patients, but examined index BCs collected on inpatient wards.^4^ Heriot *et al.* adopted a similar approach, but limited inclusion to patients with neutropenic sepsis.^19^ Those studies found a repeat BC rate of 31.6% and 74.9% respectively, compared to our early follow-up BC rate of 24.7% for the entire cohort, and 45.5% for neutropenic patients.^4,19^

Several studies examining repeated BC in known bacteremia do not note the rate of new pathogen growth.^11,21,25,26^ Our new pathogen rate of 0.7% in all BCs collected subsequent to ED workup is comparable to some studies that demonstrated new pathogens in 0–1.7% of repeat BCs,^15,19^ though some report a new pathogen rate as high as 10.1%.^27^ These studies include index and repeat BCs collected over a longer time frame than our study; in one instance the median time to identification of bacteremia was 9 days.^15^ New pathogen identification in these cases may represent infections acquired during the hospital stay. Our findings do not identify new pathogens in BCs obtained after the first repeat collection, and so the incidence of nosocomial bacteremia was low.

Several studies have examined the utility of repeating BC collection in patients with known Gram-negative bacteremia; they have varied widely in their findings and recommendations. While some found no mortality benefit associated with repeated collection,^10,16,21,25^ other authors have described an association between repeat BC collection and decreased mortality in patients with Gram negative bacteremia.^11,27,28^ Studies that describe an associated mortality benefit stress the importance of repeating BCs to alert clinicians to the possibility of an uncontrolled source of infection.^11,27^ It has long been accepted that source control is a main tenet of sepsis management and should be pursued early.^3,17,21^ It must be highlighted that the cohorts of these studies are markedly different to ours. Giannella *et al.* in particular outlines vastly different demographics than those of our study groups and only includes patients with known bacteremia.^27^ In that Italian study, the cohort without repeat BC collection was older than the group who had BCs repeated (70.2 ± 16.4 compared to 62.3 ± 16.4, *p* < 0.001 in our cohort), and had a higher Charlson Comorbidity Index (6.2 ± 2.8 compared to 5.4 ± 2.8, *p* < 0.001 in our cohort).^27^

Aside from “routine” triggers of repeat BCs in our patient cohort, the most frequent trigger for recollection was fever, and this has been demonstrated by other authors.^19^ Linsenmeyer’s 2016 study showed no association between fever and positive BCs, however “collect BCs if patient spikes fever” is a common notation in patient charts.^29^ While the fever trigger in our study returned the highest number of new pathogens, the positivity rate was low (1.0%). Previous studies reviewing factors for persistent bacteremia show a varied association with fever.^15,25^

Quality of ED BCs (indicated by single set and false positive rates) and the impact this has on the decision to repeat the test has not been examined previously. An ED BC single set rate of 21.5% in the group without early follow-up BCs reinforces a previous finding that associated single ED BC set collection with lower acuity.^30^ The higher than baseline ED BC false positive rate in the early follow-up BC group (2.7%) suggests that treating teams sought to clarify the relevance of equivocal ED BC results.^2^ Improving ED BC collection practices may thus decrease the need for repeated collection in these cases.

Cohort and result variability in studies examining BC practices demonstrates a lack of cohesion in clinical practice. Practice is variable even within our center–BCs are not universally repeated, and clearance of bacteremia is not always confirmed. There are no local guidelines that dictate practice. Aside from the management of *S. aureus* bacteremia, there are few resources to guide clinical decision making around BC recollection.^31^ These factors suggest that while our findings may not be generalizable to all health care contexts, local reviews of BC practices are advisable.

Whether “routine,” triggered by deterioration or fever, or to clarify a known positive result, collection of additional BCs within 48 hours of ED investigation for bacteremia is unlikely to be of benefit to patients. Thorough ED workup, including quality BC collection and culturing of other potential infective sources is the best way to optimize pathogen identification and ensure patients receive adequate antibiotic coverage. We recommend against re-culturing within 48 hours of adequate ED BCs, regardless of the trigger. The delay to ED BC results—several hours to days by the nature of the test—is best spent ensuring that organ support, source control, and empiric antibiotic cover is adequate.

As it is beyond the scope of this inquiry, future work will aim to elucidate the clinical significance (or insignificance) of persistent Gram-negative bacteremia, identify clinical characteristics associated with persistent bacteremia, and determine the utility of screening bacteremic patients for negativity. A trial that randomizes participants to repeat BC collection <48 hours versus >48 hours after ED workup may be contributory. It may also be of benefit to include health economics analyses in any future work of this nature.

## Data Availability

The datasets used and analyzed during the current study are available from the corresponding author on reasonable request.
